# Virtual simulation of the biomechanics of the abdominal wall with different stoma locations

**DOI:** 10.1038/s41598-022-07555-z

**Published:** 2022-03-03

**Authors:** Lluís Tuset, Manuel López-Cano, Gerard Fortuny, Josep M. López, Joan Herrero, Dolors Puigjaner

**Affiliations:** 1 Departament d’Enginyeria Informàtica i Matemàtiques, Universitat Rovira i Virgili, Av Països Catalans 26, Tarragona, Catalunya Spain; 2 Abdominal Wall Surgery Unit, Department of General Surgery, Hospital Universitari Vall d’Hebron, Universitat Autònoma de Barcelona, Barcelona, Spain; 3 Departament d’Enginyeria Química, Universitat Rovira i Virgili, Av Països Catalans 26, Tarragona, Catalunya Spain

**Keywords:** Mathematics and computing, Gastrointestinal diseases, Biomedical engineering

## Abstract

An ostomy is a surgical procedure by which an artificial opening in the abdominal wall, known as a stoma, is created. We assess the effects of stoma location on the abdominal wall mechanics. We perform three-dimensional finite element simulations on an anatomy model which was generated on the basis of medical images. Our simulation methodology is entirely based on open source software. We consider seventeen different locations for the stoma incision (trephine) and we simulate the mechanical response of the abdominal wall when an intraabdominal pressure as high as 20 kPa is applied. We focus on factors related to the risk of parastomal hernia development such as the deformation experienced by the abdominal wall, the stress levels supported by its tissues and the corresponding level of trephine enlargement. No significant dependence was found between stoma location and the levels of abdominal wall deformations or stress supported by tissues, except for the case with a stoma located on the linea alba. Trephine perimeter and area respectively increased by as much as $44\%$ and $85\%$. The level of trephine deformation depends on stoma location with considerably higher trephine enlargements found in stomas laterally located with respect to the rectus abdominis muscle.

## Introduction

An ostomy is the creation of an artificial opening into the abdominal wall (AW) through which bodily waste is rerouted outside of the body. This procedure is used to treat certain diseases related to the digestive or urinary tract. The opening created is called stoma and it is placed on the abdominal wall. One of the most common complications of ostomies is the appearance of a parastomal hernia (PH) after the construction of the stoma. Even though PH is a very frequent problem (with reported incidences as high as $78\%$^1^ or $86\%$^2^ when identified by computed tomography) it is difficult to treat and has a huge impact on the quality of life of patients who suffer from it^3–6^. In recent years, there has been a striking interest in the prevention of PH with prosthetic meshes^7–13^. However, advantages of a prosthetic mesh remain a matter of debate^14,15^.

Preventive surgical practices of PH other than the placement of a mesh have been described. The use of a stoma incision (trephine) of a diameter not larger than 25 mm was suggested under the assumption that PH were unlikely to develop for a small trephine provided that its size did not increase with time^16^. However, a more recent study showed that enlargement of the trephine, regardless of its original size, took place in almost every surveyed ostomy patient and that most patients developed PH even though the median trephine diameter of the patient cohort was below 25 mm^2^. Other surgical practices, such as an extraperitoneal exteriorization of the stoma (extraperitoneal tunneling of the stoma between the peritoneum and the AW) have been suggested for the prevention of PH. Although these practices were supported by some promising results most of the data came from observational studies, making extraperitoneal exteriorization of the stoma a controversial option^17^.

In a recent systematic review, no robust clinical data have been found to indicate which location of the stoma into the AW is optimal for PH prevention^18^. Three-dimensional (3D) modelization of the AW could be of great help to appreciate dynamically and objectively the influence of anatomic variations on the functioning of the AW^19,20^. Numerical simulations can complement clinical studies and can provide data that help abdominal surgeons in their decisions. In the present study, we applied the finite element (FE) computational setup previously developed by Tuset et al.^21^ to investigate the effect of different transperitoneal stoma locations on the biomechanical behavior of the AW. We considered seventeen different locations for stoma incisions and we focused our analysis in two aspects. First, we assessed whether the response of the AW to a certain level of intraabdominal pressure (IAP), measured in terms of both the deformation and stress levels experienced by the tissues, is affected by the presence of a stoma. In addition, we analyzed the corresponding enlargements and deformations experienced by the trephine. Enlargement of the stoma incision is generally considered a risk factor for PH, even though statistical analyses of patient data on the subject might be inconclusive^2^.

## Methods

### Geometry model

**Figure 1:**
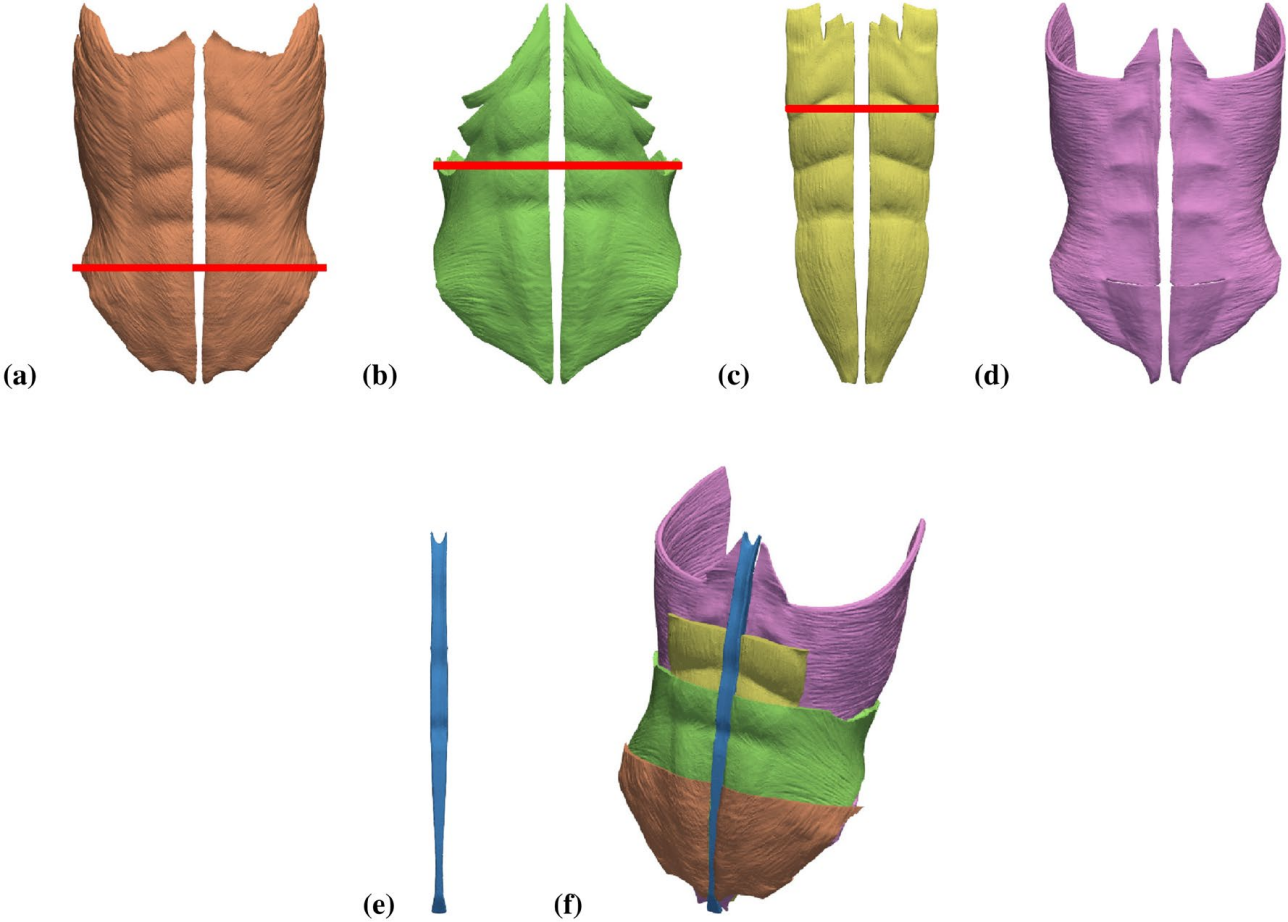
Geometry model of the abdominal wall. The elements included in the model are: (**a**) right and left external oblique muscles (EO), (**b**) right and left internal oblique muscles (IO), (**c**) right and left rectus abdominis muscles (RA), (**d**) right and left transverse abdominis muscles (TR), and (**e**) linea alba (LA). (**f**) View of the whole model; note that muscle regions above the red lines in parts (**a**)–(**c**) were removed from the plot and that since the muscles are superimposed the surfaces of the remaining (non-removed) muscle regions are only partially visible in the composed image.

Figure 1 illustrates the geometry model of the AW used in the current study. The model comprises four pairs of superimposed muscles: the two external oblique muscles (EO, Fig. 1a), the two internal oblique muscles (IO, Fig. 1b), the two rectus abdominis muscles (RA, Fig. 1c), the two transverse abdominis muscles (TR, Fig. 1d) and the linea alba (LA, Fig. 1e). Figure 1f shows how the four sets of involved muscles are superimposed in the geometry model.

Our geometry model was based on computerized tomography (CT) images available in the BodyParts3D database for anatomy^22^. Three-dimensional triangular surface meshes for each of the individual elements in the model were downloaded from this database and were subsequently refined. The refined meshes were then merged and undesirable intersections between contiguous elements were removed in order to obtain physically consistent surface meshes. These surface meshes, with a total of 892, 283 triangles, were then uploaded into the Gmsh open source software^23^ where the corresponding 3D volume meshes were built. Finally, the individual volume meshes were compounded into a global computational mesh consisting of 3, 495, 765 tetrahedra.

**Figure 2:**
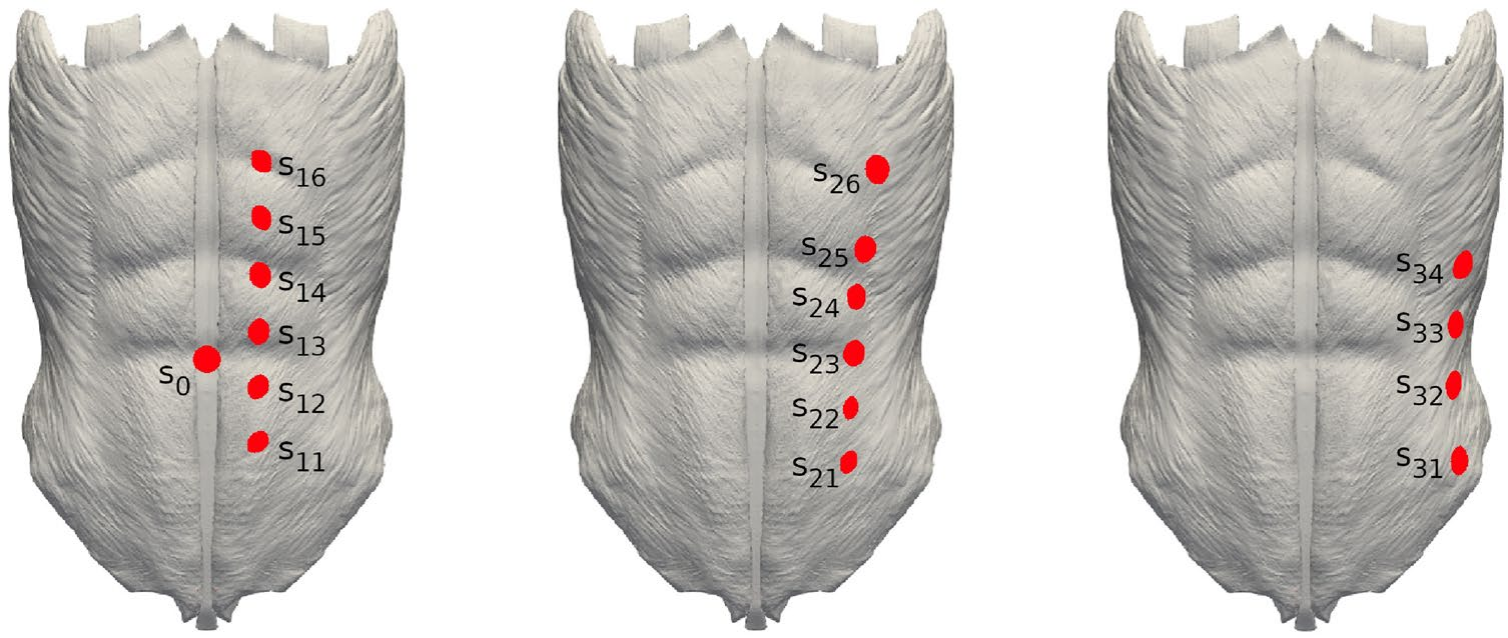
Trephine locations (colored in red) on the outermost AW surface and their labels.

In the present study, the stoma incision was modeled as a circular cylindrical orifice of diameter 2 cm. We considered 17 different locations for the stoma distributed over the AW, as shown in Fig. 2. Except for one stoma located on the LA, stomas are distributed along three vertical lines on the left side of the AW. The stoma located on the LA is denoted as $s_0$ and the rest of stomas are labelled as $s_{i,j}$, where the *i* index denotes the particular vertical line (with *i* growing with increasing horizontal distance to the vertical midline) and *j* denotes the vertical position of the stoma in the *i*th vertical line (with *j* increasing with height).

### Material properties

We assumed a linear elastic behavior of the AW tissues,

1 $$\begin{aligned} \varvec{\sigma } = E \varvec{\varepsilon }, \end{aligned}$$

where the stress tensor ($\varvec{\sigma }$), characterizing the force per unit surface area experienced by a material volume, is proportional to the strain tensor ($\varvec{\varepsilon }$), whose components represent the relative deformations in the material volume along each spatial direction. To describe the mechanical behavior of a linear elastic material two parameters are required, the Young’s modulus *E*, which provides the stiffness of the material (as can be seen in Eq. (1), for a given $\varvec{\sigma }$ level the larger the *E* value the smaller the relative deformation, $\varvec{\varepsilon }$) and the Poisson’s ratio ($\eta$), which measures the relative volume change as a result of the deformation.

In the present study, the values of *E* for the EO, IO, RA and TR muscles were prescribed following the experimental uniaxial tensor tests performed by Cardoso^24^. The specific *E* values for each tissue are listed in Table 1. For the LA, which is essentially a tendinous tissue and thus the stiffest one in the AW, we used the $E=72$ MPa value reported by Cooney et al.^25^. Note that the present model does not include soft tissues such as skin or subcutaneous abdominal fat. The underlying hypothesis is that as the stiffness of soft tissues is at least one order of magnitude smaller (lower *E*) than the stiffness of the AW muscles^26^ then muscles are the tissues actually determining the biomechanical response of the AW. All the involved tissues were characterized with a Poisson ratio of $\eta =0.3$.

**Table 1:** Values of Young’s modulus (*E*) assumed in the present study for the different tissues in our AW model.

Tissue	*E* (MPa)	References
EO	1	Cardoso^24^
IO	0.65	Cardoso^24^
RA	0.52	Cardoso^24^
TR	1.03	Cardoso^24^
LA	72	Cooney et al.^25^

### Numerical simulation

All of the present FE simulations were carried out using the Code Aster open source software^27^. A given uniformly distributed IAP value was set for the computational domain regions corresponding to the inner AW surface. In addition, a fixed zero deformation boundary condition was set for the regions on the AW edges, where the real muscular tissue would be attached to bone tissue. For each particular geometry, we considered five levels of IAP up to $P=20$ kPa (150 mmHg), a maximum value which would be typically achieved when coughing or jumping^28^. In each simulation, the distributions of stress ($\varvec{\sigma }$) and deformations along the AW were calculated. As $\varvec{\sigma }$ is a tensor quantity, often its physical interpretation is not straightforward. The so-called von Mises stress, $\sigma _v$, was therefore calculated and analyzed. The von Mises stress is a scalar quantity devised to characterize the risk of rupture of a solid material when subjected to stresses^29^.

As mentioned earlier, trephine enlargement seems to be a key risk factor for the development of PH. Consequently, in every simulation the resulting trephine dimensions (perimeter and area) in the deformed geometry were also measured and the relative change with respect to the reference (non-deformed) geometry was calculated.

## Results

We performed FE simulations with the reference geometry model (no stoma) and with each of the 17 geometry models having a stoma in the locations shown in Fig. 2. In what follows, we analyze the effects of IAP on the overall AW mechanics and, in particular, the corresponding levels of trephine enlargement. For the sake of compactness, only results from simulations with the largest IAP value, $P=20$ kPa, are presented here.

### Deformation and stress distribution on the abdominal wall

**Figure 3:**
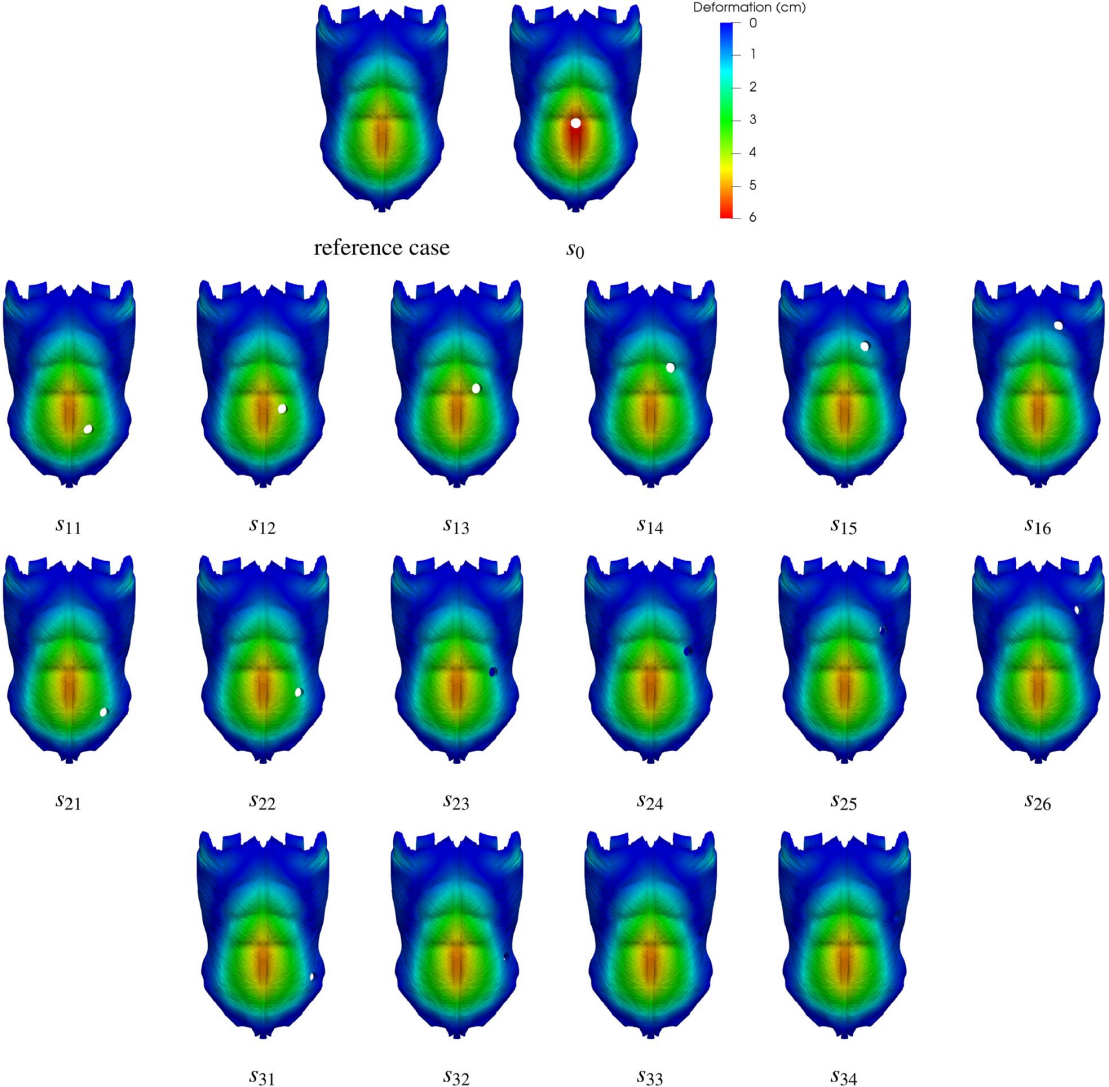
The predicted deformation distributions are plotted on the base (undeformed) geometry of the abdominal wall for all the cases investigated, that is, the reference case (no stoma) and the seventeen stoma locations. In all cases, the results correspond to the simulations with the maximum value of the applied intraabdominal pressure, $P=20$ kPa.

Figure 3 shows the deformation distribution on the outer AW surface as predicted by our numerical simulations for all the cases investigated. Note that since in this figure deformations are plotted on the undeformed geometry trephines preserve their original size (trephine enlargement will be the subject of the next subsection). As expected, the result of applying any positive IAP level is always a forwards protrusion of the AW, with a maximum deformation of 52.09 mm in the reference case. In the 16 cases with the stoma located away from the LA, predicted maximum deformations ranged between 51.09 and 53.25 mm, which indicates that differences with respect to the reference case are not significant. Moreover, as it can be observed in Fig. 3, all deformations for geometries other than $s_0$ strongly resemble the distribution for the reference case. Similarly, small differences were found between all cases other than $s_0$ in terms of the von Mises stress distributions, with maximal values ($\sigma _v^{max}$) in the 11.19–11.36 MPa range (with $\sigma _v^{max}=11.29$ MPa in the reference case).

**Figure 4:**
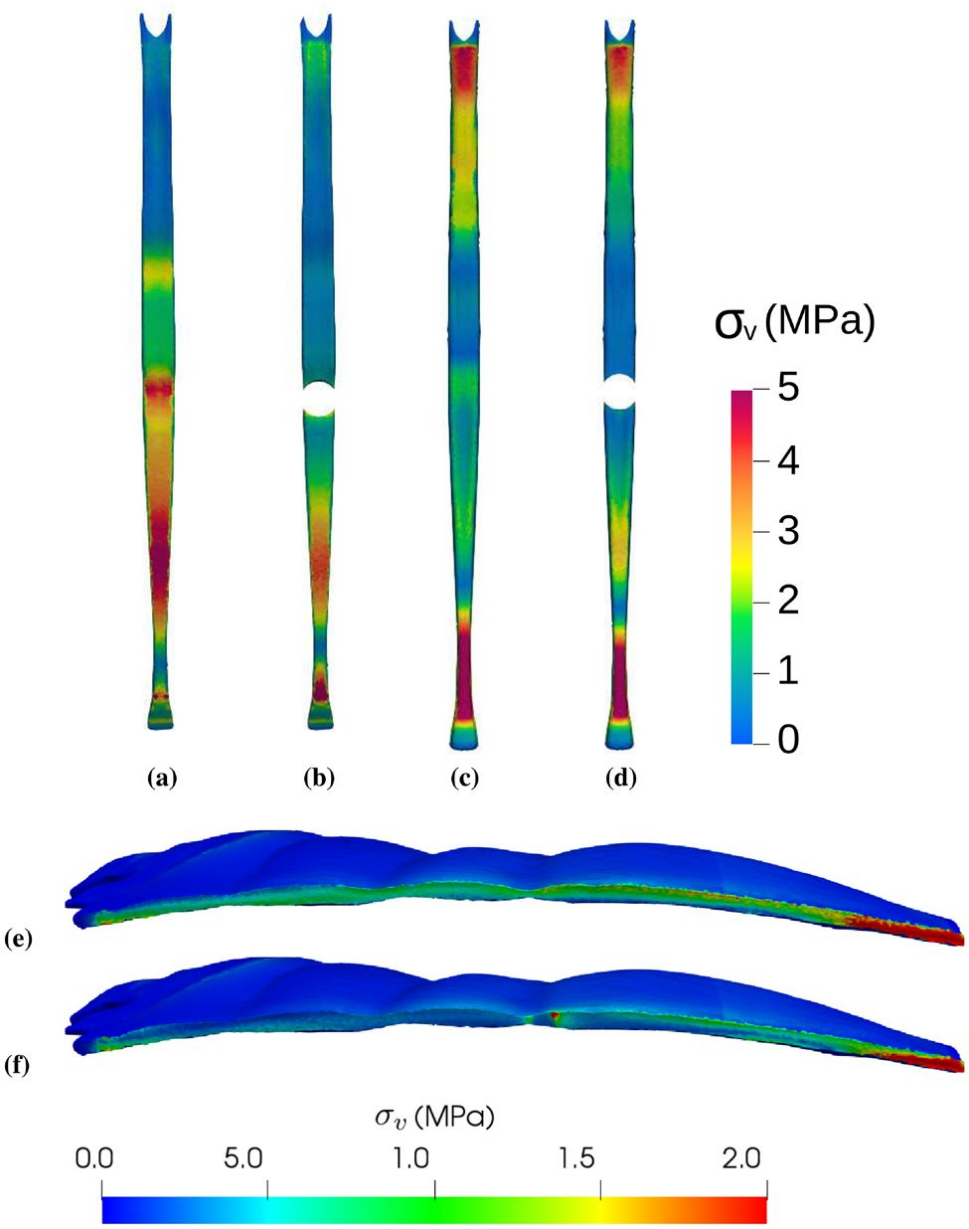
Distribution of Von Mises tension ($\sigma _v$) on the anterior (**a**,**b**) and posterior (**c**,**d**) surfaces of the linea alba and (**e**,**f**) on the inner lateral region of the left rectus abdominis muscle (the surface that is in contact with the LA). (**a**,**c**,**e**) Reference case (model without incisions). (**b**,**d**,**f**) Case with a stoma located on the linea alba ($s_0$). Note that in parts (**e**) and (**f**) the axes are rotated so that the leftmost region of the RA here corresponds to the uppermost RA region in Fig. 1c.

The $s_0$ case was devised as an *acid test* for the current methodology. That is, the classical recommendation that a stoma should not be constructed through a midline laparotomy incision^1^ ought to be also supported from a mechanical standpoint. As illustrated in the first row of Fig. 3, the maximum AW deformation is about a $18\%$ larger in the $s_0$ case (61.23 mm) than it is in the reference case (52.09 mm). The corresponding $\sigma _v^{max}$ increase is more modest, about a $3\%$ ($\sigma _v^{max}=11.65$ MPa for $s_0$). The LA plays a key role in AW mechanics as it pulls together the muscles from the left and right sides (see Fig. 1f). Consequently, as shown in Fig. 4a,c, in the current IAP-promoted AW expansion scenario LA is the element experiencing the highest stress levels, with the largest $\sigma _v$ values found near its lower edge (closer to the pubic region). From a mechanical point of view, the main drawback with the $s_0$ stoma location is that the trephine severs the LA into two independent sections (see Fig. 4b,d). Despite the larger AW deformation for $s_0$ in Fig. 3, comparison of Fig. 4d with Fig. 4b shows that the two independent LA sections keep performing their main function, i.e., they remain attached to the muscles from both sides of the AW.

As the two rectus abdominis are the muscles in closer contact with the LA through their inner lateral surfaces, one would expect AW expansion to produce relatively high stress levels within the RA tissue. Figures 4e,f show the $\sigma _v$ distribution on the inner lateral region of the left RA muscle for the reference case and the $s_0$ case. In the reference case (Fig. 4e), the largest $\sigma _v$ values are concentrated at the lower part of the muscle (rightmost section of the plot), consistently with the stress distribution on the LA itself (see Fig. 4c). In contrast, in the $s_0$ case (Fig. 4f) a second region of high $\sigma _v$ (i.e., a red spot) appears around the axial location of the trephine.

### Trephine enlargement

**Figure 5:**
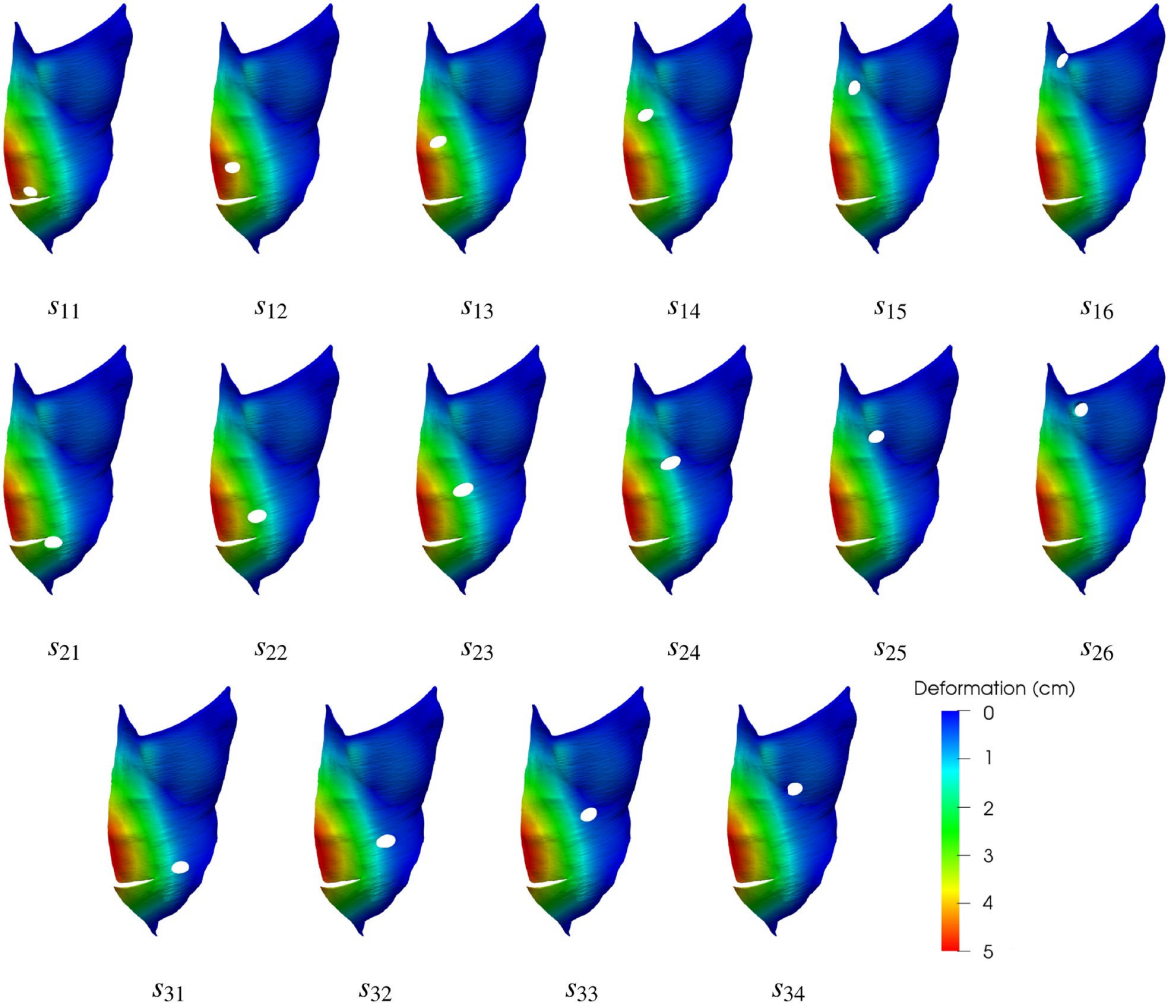
Deformed geometries in the cases where the enlargement of the stomas was apparent at first sight when an IAP of $P=20$ kPa was applied. For the sake of clarity, only the left TR muscle is shown in each case. The deformation distribution on the TR surface is represented by color levels, as specified in the accompanying color box.

The trephine original size was significantly increased as a result of the AW deformation in all the cases investigated, as illustrated in Fig. 5 where the deformed geometry of the left TR muscle is displayed. The elliptic forms adopted by stoma incisions in the deformed geometries are clearly visible in these plots. In agreement with the radiological measurements reported by Ho et al.^2^, our simulations predict that the trephine sagittal diameter grows more than its axial diameter does. Table 2 summarizes the deformations experienced by stomas in terms of trephine perimeter and area, as well as the corresponding percent increase of these two quantities with respect to the original geometry. Note that when the largest IAP of 20 kPa is applied trephine enlargements as high as $44\%$ in terms of perimeter and $85\%$ in terms of area (for the $s_{32}$ stoma) are obtained. A topic which is the subject of some controversy in the literature is whether trephine enlargment levels depend on stoma location. More particularly, the question is whether or not stomas located lateral to the left RA muscle tend to experience largest trephine enlargements than do stomas crossing the RA. For the latter type of stomas, we can see in Table 2 (the ones labeled as $s_{1,j},\,1\le j \le 6$; see also Fig. 2) that the average incrase in trephine area is $28\%$ whereas for the stomas lateral to the RA muscle the corresponding increases are $53\%$ ($s_{2,j}$) and $54\%$ ($s_{3,j}$). Thus, the present simulations predict trephine enlargements that, on the average, are more pronounced for stomas located lateral to the RA muscle. This result is in apparent contradiction with the findings by Ho et al.^2^, who found no statistically significant relation between the rate of trephine size progression and stoma position. Notwithstanding, these authors acknowledged as a limitation of their data analysis the fact that only $11.7\%$ of patients had a stoma created lateral to the RA muscle.

**Table 2:** Area and perimeter of stomas in the deformed geometry when an intraabdominal pressure of $P=20$ kPa is applied. The percentage of increase with respect to the undeformed geometry is also included. Note that in the undeformed geometry the diameter of the stoma is 2 cm, the area is therefore $3.14\ \hbox {cm}^2$ and the perimeter is 6.28 cm. In all cases, the dimensions of the deformed trephines were measured at the innermost surface of the abodminal wall.

Stoma	Area of the deformed stoma ($\hbox {cm}^2$)	$\%$ increase in area	Perimeter of the deformed stoma (cm)	$\%$ increase in perimeter
$s_0$	3.76	20	6.93	10
$s_{11}$	3.59	14	6.88	9
$s_{12}$	3.64	16	6.96	11
$s_{13}$	4.18	33	7.56	20
$s_{14}$	4.18	33	7.46	19
$s_{15}$	4.25	35	7.45	19
$s_{16}$	4.07	30	7.30	16
$s_{21}$	4.75	51	7.76	23
$s_{22}$	4.82	53	8.05	28
$s_{23}$	5.37	71	8.70	38
$s_{24}$	5.58	78	9.00	43
$s_{25}$	4.34	38	7.56	20
$s_{26}$	3.93	25	7.14	14
$s_{31}$	4.28	36	7.50	19
$s_{32}$	5.82	85	9.05	44
$s_{33}$	4.34	38	7.59	21
$s_{34}$	4.91	56	8.09	29

## Discussion

Our study shows that the creation of a stoma, unless its location is poorly chosen, does not compromise the mechanical consistency of the AW when subjected to IAP levels as high as 20 kPa. More precisely, except for the case with a stoma located on the LA ($s_0$), the amount of deformation of the AW (visualized as a forward protrusion of the patient’s belly) and the stress levels that it supports show a very weak dependence on stoma location.

On the other hand, an augment of the trephine size was observed in all of the simulations. Such a trephine enlargement was measured in terms of the increase in both trephine perimeter and cross-area. Our simulations therefore indicate that trephine enlargement is inherent to the AW deformation that results when a certain IAP level is applied. It seems plausible to suggest that as trephine enlargement is related to PH development^2^ then parastomal hernia would be a long run complication inherent to the ostomy procedure. Notwithstanding, it is important to place the present results in the proper scope. Our model predicts short-time trephine enlargements that would be completely reversible. That is, the whole AW and thus the trephines would recover their original shape once the IAP is released. What is seen and measured in the patients’ CT scans^2^ is trephine defect, i.e., the level of permanent (irreversible) enlargement of trephines. We can think of trephine defect in terms of either material fatigue, micro-lesions or, more generically, a progressive adaption of biological tissues to the surrounding constraints. In an ideal world permanent trephine enlargement should not occur but reality (clinical findings) is that trephine defect increases with time after stoma creation. We cannot presently predict, for example, whether trephine defect would develop faster in a (hypothetical) patient with a quiescent life style, who might however experience occasional large AW deformations (e.g., a sudden high peak in IAP level), or in a second patient undergoing mild but frequent exercising.

The present simulations also show that stomas placed lateral to the RA muscle experience higher trephine enlargements as a result of an IAP, a fact suggesting that creation of laterally placed stomas ought to be avoided. However, this result has to be interpreted with caution because of the aforementioned difference between the ideal (reversible) trephine enlargement, predicted in our simulations, and the real (irreversible) trephine defect experienced by ostomy patients. Future research, which will hopefully include radiological measurements and data analyses in large ostomy patients’ cohorts^30^, will no doubt shed more light on the subject. Moreover, our methodology could be used in the future to assess the risks associated to trephine enlargement in a patient-specific basis. For this purpose, simulations would be carried out in geometries built from the patient’s CT scans.
